# Liver Transplantation for Acute Liver Failure due to Mushroom Poisoning

**DOI:** 10.5152/tjg.2024.24226

**Published:** 2024-11-04

**Authors:** Hayri Canbaz, Attila Bestemir, Sami Akbulut, Sezai Yilmaz, Yusuf Yavuz

**Affiliations:** 1 Department of Emergency Medicine, Yıldırım Beyazit University, Yenimahalle Training and Research Hospital, Ankara, Türkiye; 2 Department of Private Health Institutions with Outpatient Diagnosis and Treatment, Ministry of Health, Ankara, Türkiye; 3 Department of Surgery and Liver Transplant Institute, İnonu University Faculty of Medicine, Malatya, Türkiye; 4 Department of General Surgery, Necmettin Erbakan Universitesi Meram Faculty of Medicine, Konya, Türkiye

**Keywords:** Liver transplantation,, acute liver failure,, mushroom poisoning,, prognosis

## Abstract

**Background/Aims:**

Liver transplantation is a life-saving approach in some cases of mushroom poisoning, which is one of the important causes of acute liver failure. However, debate continues regarding the timing of liver transplantation. The aim of this study is to retrospectively evaluate the results of patients who underwent liver transplantation due to mushroom poisoning.

**Materials and Methods:**

In this descriptive and observational study, the demographic and clinical data of 26 patients who presented to emergency units due to clinical features of acute hepatic failure secondary to mushroom poisoning between October 2008 and November 2023 and who underwent emergent liver transplantation were retrospectively reviewed.

**Results:**

A total of 26 patients with a median (IQR) age of 39 (36) years were included in this study. The patients were divided into two groups: alive (n = 18) and dead (n = 8). No statistically significant differences were found between groups in terms of age, BMI, blood groups, hepatic encephalopathy grade, biochemical analysis obtained on the first days of hospital admission (AST, ALT, creatinine, ammonia, PTT, INR, albumin, platelets, HGB), ICU stay, cold ischemia time (CIT) and warm ischemia time (WIT), total bilirubin (*P* = .052), and time from poisoning to admission (*P* = .051). On the other hand, there were statistically significant differences between the alive and dead groups in terms of MELD score (*P* = .016; 23 vs. 34), re-transplantation (*P* = .022; 0% vs. 37.5%), hospital stay (*P* = .004; 24 vs. 6 days), and follow up (*P* < .001; 3423 vs. 5 days).

**Conclusions:**

This study showed that mortality was higher in patients with high MELD scores and patients who underwent re-transplantation. However, this study needs to be supported by multicenter prospective studies.

**Main Points**

- Acute liver failure is defined as severe acute liver injury of shorter than twenty-six weeks’ duration with encephalopathy and impaired synthetic function (INR ≥ 1.5) in a patient without cirrhosis or preexisting liver disease.
- Liver transplantation is a life-saving, curative treatment for patients exposed to mushroom poisoning.
- There is no clear consensus on which patients and when liver transplantation should be performed.
- Timely referral of patients to experienced centers is very important in terms of patient management and decision-making.

## Introduction

The most common causes of acute liver failure, which is characterized by liver dysfunction, neurological dysfunction, and coagulopathy, are drug-induced hepatotoxicity, viral hepatitis, Wilson’s disease, Budd-Chiari Syndrome, and mushroom poisoning.^1^ The Amanita phalloides is considered one of the most perilous mushrooms, as it is responsible for the majority of fatal cases of mushroom poisoning in humans worldwide. Among approximately 100 000 types of mushrooms worldwide, about 100 are poisonous to humans and can develop a clinical syndrome of intoxication.^2^ In most instances, the clinical scenario starts with gastrointestinal tract symptoms, followed by acute hepatic decompensation and multiple organ failure, which could ensue, with a recorded mortality rate ranging between 4.8% and 34.5%.^3,4^

Acute liver injury secondary to mushroom poisoning has been managed by detoxifying agents like N-acetylcysteine, silymarin, and penicillin.^3^ Supportive treatments with activated charcoal have been reported as well.^3^ In more severe situations, plasmapheresis or extracorporeal liver support has been adopted.^3,5^ When the clinical situation ends up with acute liver failure, manifested by progressive liver dysfunction, neurological impairment, and coagulopathy emergency liver transplantation (LT) may be a life-saving necessity.^3,6^

However, LT is an ultra-major procedure with numerous logistics and officials that are not readily available in all regions or medical centers, as well as its overwhelming expenses. In addition, the possible complications and the need for life-long immunosuppression are among the drawbacks of LT in this clinical setting. In essence, it has been admitted that emergent LT is associated with lower survival rates compared to LT in the elective setting practiced for various chronic liver diseases.^7^ In this retrospective study, we presented the outcome of acute liver failure cases secondary to mushroom poisoning that were subjected to emergency LT in Liver Transplant Institutions in Türkiye.

## Materials and Methods

In this descriptive study, the demographic and clinical data of 26 patients who presented to emergency department throughout Türkiye with the clinical features of acute hepatic failure secondary to mushroom poisoning between October 2008 and November 2023 and who underwent emergency LT after fulfilling national criteria (mentioned later) were retrospectively reviewed. Patients with lacking medical records, patients <18 years old age, and patients who presented to emergency units with hepatic injury secondary to causes other than mushroom ingestion were excluded from the study. Age (years), gender (male and female), body mass index (BMI; kg/m^2^), patient blood group (O, A, B, AB), presence and level of hepatic encephalopathy (grade I-II-III-IV), type of transplantation (deceased or living donor LT), duration of stay in the intensive care unit, graft type of the patients registered in the Turkish Ministry of Health data system, graft loss, cold ischemia time (hours), warm ischemia time (minutes), post-operative complications and follow-up (days) period after emergent LT were reviewed. In addition, partial thromboplastin time (PTT; seconds), international normalized ratio (INR), albumin (g/dL), creatinine (mg/dL), aspartate aminotransferase (AST; U/L), alanine transaminase (ALT; U/L), gamma glutamyl transpeptidase (GGT; IU/L), alkaline phosphatase (ALP), ammonia (ug/dL), white blood cells (WBC; 103/uL), platelet (103/uL) and hemoglobin (HGB; g/dL) values, which were obtained during their first admission to the hospital and before discharge or death after emergency LT were examined. The model for end-stage liver disease (MELD) score was calculated for each case using pocket program (https://www.mdcalc.com/calc/10437/model-end-stage-liver-disease-meld). Since we did not have any patients with a MELD score between 1 and 10 points, they were not included in the grouping.

### Definition of Acute Liver Failure

Acute liver failure is defined as severe acute liver injury of shorter than twenty-six weeks’ duration with encephalopathy and impaired synthetic function (INR ≥ 1.5) in a patient without cirrhosis or preexisting liver disease.^8^ The diagnosis of acute liver failure induced by mushroom poisoning was based on: (i) the recent ingestion of wild mushrooms associated with watery diarrhea, vomiting, and/or abdominal pain within 24 hours after ingestion; (ii) the clinical and laboratory criteria of acute liver failure; and (iii) the absence of any other cause for acute liver failure.^9^

### Criteria for Emergent LT

The decision for emergent LT was based on King’s College criteria for non-acetaminophen causes. These criteria are as follows: INR > 6.5 or presence of three of the following five following criteria: (i) age (≤10 or >40 years), (ii) certain etiology (non-A, non-B viral hepatitis, and other drugs), (iii) interval between jaundice and encephalopathy (>7 days), (iv) INR > 3.5, (v) serum total bilirubin level (>17.4 mg/dL).^10^

### Study Protocol and Ethics Committee Approval

This study involved human participants and abided by the ethical standards of the institutional and national research committee, as well as the 1964 Helsinki Declaration and its later amendments or comparable ethical standards. Ethical approval was obtained from the institutional review board (IRB) of Ankara City Hospital for Non-Interventional Clinical Research (approval no: 2022/ E2-22-18844; date: May 27, 2022).

### Statistical Analysis

IBM SPSS Statistics software version 25.0 (IBM SPSS Corp.; Armonk, NY, USA) was used for this study. Qualitative variables were given as numbers (percentage), while quantitative variables were given as median and interquartile range (IQR = Q3-Q1). Mann–Whitney U-test was used to compare quantitative data, and Chi-square test was used to compare qualitative data. *P* < .05 was considered statistically significant.

## Results

### General Evaluation of the Study Group

Twenty-six patients diagnosed with acute liver failure secondary to mushroom poisoning who underwent emergency LT were included in the study. Twelve (46.2%) of the patients were male and the remaining 14 (53.8%) were female. The median (IQR) age, BMI, MELD, from poisoning to hospital admission, ICU stay, hospital stay, CIT, and WIT were 39 (36) years, 25 (8) kg/m^2^, 25 (14) points, 6 (21) days, 4 (4) days, 18 (23) days, 3 (6) hours, and 48 (15) minutes, respectively.

### Subgroup Analysis

Patients were divided into two groups: alive (alive group; n = 18; 69.2%) and dead (dead group; n = 8; 30.8%). Due to the nature of mushroom consumption, there were no cases of mushroom poisoning during the winter season. The median (IQR) age of the dead group was 53 (31) and *in alive group*, it was 38 (28) years (*P* = .196). The mortality rate was 16.7% in males and 42.8% in females but there were no statistically significant differences between groups in terms of gender (*P* = .216). The median (IQR) BMI was 24 (5) for the alive group and 26 (10) for the dead group (*P* = .701). The median (IQR) MELD score was 23 (11) points for the alive group and 34 (8) points for the dead group and statistically *significant differences were* found between groups in terms of MELD scores (*P* = .016).

Deceased donor liver transplantation was performed in 14 (53.8%) patients, and LDLT was performed in 12 (46.2%) patients. Twelve DDLT patients (85.7%) received a whole liver graft, and remaining two received a split liver graft. Regarding blood group, 17 (65.4%) were blood group A, 5 (19.2%) were blood group O, 3 (11.5%) were blood group B, and 1 (3.8%) was group AB. The rate of patients with Rh (+) was found to be 84.6%, and the rate of patients with Rh (−) was 15.4%.

The median (IQR) CIT was 3 (6) hours for the alive group and 5 (8) hours for the dead group (*P* = .528). The median (IQR) WIT was 45 (16) minutes for the alive group and 50 (15) minutes for the dead group (*P* = .964). The median (IQR) ICU stay was 4 (4) days for the alive group and 5 (6) days for the dead group (*P* = .834). The median (IQR) hospital stay was 24 (22) days for the alive group and 6 (6) days for the dead group (*P* = .004). Since patients who develop mortality in the early postoperative period, the hospital stay is very low as expected in the dead group. While none of the patients in the alive group were re-transplanted, three of the patients in the dead group *were re-transplanted* (*P* = .022). The indication for re-transplantation in all these patients was postoperative primary graft dysfunction. There was no difference in the degree of hepatic encephalopathy between the alive and dead groups (*P* = .401).

There were no statistically significant differences between the alive and dead groups in terms of AST (*P* = 1.000), ALT (*P* = .928), total bilirubin (*P* = .052), Creatinine (*P* = .383), Ammonia (*P* = .278), PTT (*P* = .928), INR (*P* = .591), Albumin (*P* = .976), Platelets (*P* = .888), and HGB (*P* = .541) levels on the first days of hospital admission. Details were summarized in Table 1.

## Discussion

Liver transplantation is considered the biggest development in the management of numerous etiologies of acute liver failure in the last years. Though emergent LT has inferior survival rates compared to elective LT, a 1-year survival rate of as much as 80% can still be achieved in the emergency setting LT. Early identification of acute liver failure that is not responding to medical treatment and could otherwise benefit from LT is crucial in the decision-making process of LT in the emergency setting. In this study, we managed to identify those early determinants of the indication of emergency LT.

We found that the mortality rate among female patients undergoing emergent LT was double the rate among male patients, though the rate of emergent LT procedures was equal among both genders. This is somewhat different from a recent study that found a similar mortality rate among both genders, though male patients were more often subjected to emergency LT.^11^ Another 10-years study also reported a higher rate of emergent LT among male patients (from 59.4% to 62.6%) presenting with acute liver failure.^12^ It is obvious that this situation, which does not show statistical significance but shows a significant difference proportionally, needs to be supported by other clinical studies. In our opinion, many factors such as the retrospective nature of the study, the small sample size, and the absence of a control group without liver transplantation despite having mushroom poisoning may affect this situation.

Data extracted from the United network for organ sharing (UNOS) and the European liver transplant registry (ELTR) showed worse outcomes in those recipients aged 50 and over.^13,14^ Our results were aligned with this finding. Around 60% of mortalities among our recipients were over 50 years old. We concur with the point that old age is associated with worse outcomes in the emergency LT. In our study, it was found that 50% of the cases who underwent emergency LT presented to emergency department with mushroom poisoning during the autumn season. Interestingly, the same finding had been reported in an epidemiological study of mushroom poisoning in USA.^15^

It has been mentioned that the 1-year survival rate among emergency LT recipients is lower compared to chronic liver disease recipients who underwent surgery under elective circumstances.^16,17^ However, it should be noted that the survival rates for acute liver failure patients who underwent emergency LT have improved significantly over the last three decades, with estimated 1- and 5-year survival rates of 79% and 72% reported from a European study. Another study from the USA reported 1- and 5-year survival rates of 85% and 69%, respectively.^18^ In a recent study from Türkiye Ertugrul and colleagues,^19^ the 1- and 5-year overall survival rates of 13 patients who underwent LDLT due to acute liver failure were 84.7% and 69.3%, respectively. In our cohort, the 1- and 5-year overall survival rates were 69.2% and 69.2%, being lower than those reported in the aforementioned studies. This could be explained by the fact that all of our patients underwent emergency LT while presenting with higher grades of hepatic encephalopathy and poor general condition.

The most frequently encountered complication was post-operative disturbed consciousness and delayed neurological recovery. Similar studies showed that the most common cause of death in emergency LT patients was infection.^19,20^ We didn’t encounter infectious complications among our recipients, though. In fact, the post-operative outcome after emergency LT for acute liver failure is influenced by multiple factors.^19,20^

From a study that included 75 thousand cases and spanned 13 years, the blood groups of emergency LT recipients were as follows: blood group O (44.01%), blood group A (37.6%), and (5.02%) were blood group AB (14). Another study of a 10-year period showed that blood group O recipients constituted 49.2%, followed by blood group A (36.7%), blood group B (11.5%), and finally 2.6% were blood group AB (12). Though the small sample size of our study, our data is similar to the reported literature. We found 66.6% of our patients were blood group A, 25% were blood group O, and 8.4% were blood group B. Therefore, most acute liver failure cases in need for emergency LT were among blood group A and O. Like the reported literature, it appears that AB blood group patients were less likely to receive an emergency LT in the acute setting.^12,14^

It is well appreciated that the mere presence of hepatic encephalopathy early in the course of acute liver failure underlies a poor prognosis after emergency LT. If renal failure or another organ dysfunction ensues, the prognosis could be worse. Though the LT candidacy criteria could vary among transplant institutions, the presence of hepatic encephalopathy is one of the main criteria in the decision-making for emergency LT.^21^ In patients who progress to grade III-IV hepatic encephalopathy, the mortality rate could reach between 40% and100%.^21^ In our study, a relationship was found between advanced-grade hepatic encephalopathy and high mortality rate.

Model for end-stage liver disease score is a consistent tool for prioritizing enlisted patients for LT since 2002. It ranges between 6 and 40 with patients with higher scores being of poor prognosis.^12,18,22^ Consistent with the literature, MELD scores were found to be high in the patients who died in our study. There was one mortality in 6 patients with MELD scores between 11 and 20. One out of 8 patients with MELD scores between 21 and 30 died. Again, 6 out of 8 patients whose MELD scores were between 31 and 40 died (*P* = .017). In other words, the MELD score, which consists of bilirubin, INR, and creatinine components, is an important factor associated with mortality in acute liver failure. However, the sample size of this study is the most important obstacle to giving a strong message.

In a study by Kim and colleagues,^12^ patients with acute liver failure and a body mass index of more than 30 kg/m^2^ were noted to have worse outcome when they underwent emergency LT. In our study, we didn’t find a significant difference in BMI among recipients who died, and was not considered a risk factor from a statistical standpoint.

Coagulation impairment, namely INR and PTT rise, are an integral components of acute liver failure diagnosis. It results from acute liver insults that cause massive liver damage and associated liver dysfunction.^22^ Many studies have shown high INR and ammonia levels to be among the most important prognostic factors.^23,24^ Similarly, median ammonia values were observed to be high in the patients in our study, but this elevation did not reach to the statistical significance.

Albumin is an important surrogate of the liver’s synthetic function. Its plasma level decreases in all diseases with liver dysfunction. In mushroom poisoning, the decline in plasma albumin levels will parallel the liver damage and the degree of liver dysfunction. In addition, it is a component of the Child-Pugh score used for decades in many regions to categorize liver performance. Studies have found that a decrease in albumin levels is associated with mortality.^25^ In our study, albumin levels were not found to be a significant factor.

Alanine aminotransferase and AST are enzymes found extensively in hepatocytes. Elevations in plasma ALT and AST levels are associated with hepatocyte damage, and the degree of rise correlates with the degree of liver damage. Mushroom poisoning is associated with a massive rise in AST and ALT.^25^ Different studies have shown a poor prognosis in emergency LT recipients who underwent surgery with increased plasma levels of AST, ALT, INR, BUN, and Creatine.^12,16^ A recent study by Badsar and colleagues^26^ reviewed the laboratory results of patients with mushroom poisoning taken at the time of admission and observed that 28.4% of the patients had leukocytosis as well as a platelet count below 100 000. They also found that 5.9% of patients had elevated plasma AST levels and 9.8% had elevated plasma ALT values, and those patients had a worse prognosis.^26^ In our study, there was no statistically significant correlation between the serum level of transaminases or total bilirubin and the emergency LT outcome. It is thought that this result is due to the development of fulminant hepatitis in the patients.

Liver transplantation in acute liver failure, especially in mushroom poisoning, is a situation that requires rapid decision-making. In Western countries, the use of LDLT in acute liver failure is still a controversial issue due to many factors such as donor safety, approval process, and recipient outcomes, largely because the use of cadaveric organs is higher in Western countries. In many countries, including Turkey, there is no other option other than using living donors in more than half of the patients in emergent conditions. A systematic review published in recent years has shown that LDLT in acute liver failure provides similar results to DDLT.^27^ Another study has shown that donor complications are similar in emergency and elective liver transplantation.^28^ This study has shown that there is no difference between DDLT and LDLT procedures in acute liver failure. When the complications in living liver donors used for urgent LDLT are examined, it is seen that they are no different from those performed in elective LDLT.

This study has several limitations. Firstly, since the study is retrospective, it is difficult to access all of the patient data. In order to avoid such problems, joint working groups and software systems should be developed across the country, so that it is known which parameters to record in patients applying to health care centers with acute liver disease, thus preventing data loss. Secondly, the number of patients evaluated in the study was insufficient to put forward strong arguments. To overcome this problem, multicentric studies should be organized. Thirdly, there is no analysis regarding who and under what circumstances LT should be performed in acute liver failure. To overcome this problem, a control group should be created among patients who had mushroom poisoning and did not undergo LT in the same period, and both groups should be compared in terms of demographic and clinical parameters.

In conclusion, despite the limitations mentioned above, this study is one of the most comprehensive studies in the literature in which acute liver failure due to mushroom poisoning and liver transplantation were examined. This study showed that mortality was higher in patients with high MELD scores or patients who underwent re-transplantation.

## Availability of Data and Materials:

The data that support the findings of this study are available on request from the corresponding author.

## Figures and Tables

**Table 1.** Comparison of Patients with Mushroom Poisoning in Terms of Post-Transplant Survival Status

Parameters	Alive (n = 18)	Dead (n = 8)	*P*
Age (years) [median (IQR)]	38 (28)	53 (31)	.196
Gender (n;%)			.216
Female	8 (44.4)	6 (75.0)	
Male	10 (55.6)	2 (25.0)	
BMI (kg/m^2^) [median (IQR)]	24 (5)	26 (10)	.701
MELD [median (IQR)]	23 (11)	34 (8)	.016
Blood group (n;%)			.191
0	4 (22.2)	1 (12.5)	
A	13 (72.2)	4 (50)	
B	1 (5.6)	2 (25.0)	
AB	0 (0)	1 (12.5)	
Hepatic encephalopathy (n;%)			.401
Grade II	7 (38.9)	5 (62.5)	
Grade III	11 (61.1)	3 (37.5)	
LT Type (n;%)			1.000
LDLT	8 (44.4)	4 (50)	
DDLT	10 (55.6)	4 (50)	
Re-transplantation (n;%)			.022
Yes	0 (0)	3 (37.5)	
No	18 (100)	5 (62.5)	
AST [median (IQR)]	2823 (4356)	2124 (7377)	1.000
ALT [median (IQR)]	2705 (3577)	2511 (4718)	.928
Total bilirubin [median (IQR)]	2.8 (3.1)	5 (10)	.052
Creatinine [median (IQR)]	0.8 (0.4)	1.2 (2.1)	.383
Ammonia [median (IQR)]	387 (432)	643 (1264)	.278
PTT [median (IQR)]	48 (38)	42 (34)	.928
INR [median (IQR)]	3.9 (3.3)	4.0 (6.6)	.591
Albumin [median (IQR)]	3.4 (0.5)	3.5 (0.3)	.976
Platelets [median (IQR)]	163 (145)	172 (153)	.888
HGB [median (IQR)]	12.4 (1.9)	11.5 (2.8)	.541
From poisoning to admission (days) [median (IQR)]	5 (2)	14 (28)	.051
ICU Stay (days) [median (IQR)]	4 (4)	5 (6)	.834
Hospital Stay (days) [median (IQR)]	24 (22)	6 (6)	.004
CIT (hour) [median (IQR)]	3 (6)	5 (8)	.528
WIT (min) [median (IQR)]	45 (16)	50 (15)	.964
Follow up (days) [median (IQR)]	3423 (2043)	5 (6)	<.001

ALT, Alanine aminotransferase; AST, Aspartate aminotransferase; BMI, Body mass index; CIT, Cold ischemia time; DDLT, Deceased donor liver transplantation; HGB, Hemoglobin; ICU, Intensive care unit; INR, International normalized ratio; IQR, Interquartile range; LDLT, Living donor liver transplantation; LT, Liver transplantation; MELD, Model for end-stage liver disease; PTT, Partial thromboplastin time; WIT, Warm ischemia time.
